# Artificial Neural Network Approach for Hardness Prediction in High-Entropy Alloys

**DOI:** 10.3390/ma18204655

**Published:** 2025-10-10

**Authors:** Makachi Nchekwube, A. K. Maurya, Dukhyun Chung, Seongmin Chang, Youngsang Na

**Affiliations:** 1 Department of Mechanical Engineering, Chungnam National University, Daejeon 34134, Republic of Korea; makachinchekwube@o.cnu.ac.kr (M.N.); schang@cnu.ac.kr (S.C.); 2 Korea Institute of Materials Science, Changwon 51508, Republic of Korea

**Keywords:** high entropy alloys (HEAs), artificial neural network (ANN), hardness prediction, weight distribution

## Abstract

High-entropy alloys (HEAs) are highly concentrated, multicomponent alloys that have received significant attention due to their superior properties compared to conventional alloys. The mechanical properties and hardness are interrelated, and it is widely known that the hardness of HEAs depends on the principal alloying elements and their composition. Therefore, the desired hardness prediction to develop new HEAs is more interesting. However, the relationship of these compositions with the HEA hardness is very complex and nonlinear. In this study, we develop an artificial neural network (ANN) model using experimental data sets (535). The compositional elements—Al, Co, Cr, Cu, Mn, Ni, Fe, W, Mo, and Ti—are considered input parameters, and hardness is considered as an output parameter. The developed model shows excellent correlation coefficients (Adj R2) of 99.84% and 99.3% for training and testing data sets, respectively. We developed a user-friendly graphical interface for the model. The developed model was used to understand the effect of alloying elements on hardness. It was identified that the Al, Cr, and Mn were found to significantly enhance hardness by promoting the formation and stabilization of BCC and B2 phases, which are inherently harder due to limited active slip systems. In contrast, elements such as Co, Cu, Fe, and Ni led to a reduction in hardness, primarily due to their role in stabilizing the ductile FCC phase. The addition of W markedly increased the hardness by inducing severe lattice distortion and promoting the formation of hard intermetallic compounds.

## 1. Introduction

High-entropy alloys (HEAs) represent a new class of metallic materials that have gained considerable attention in recent years due to their unconventional alloy design and exceptional mechanical and functional properties. Unlike traditional alloys, which are typically based on one or two principal elements with minor additions, HEAs are composed of at least five principal elements in near-equiatomic proportions, typically ranging between 5 and 35 atomic percent [1]. This unique compositional strategy results in a high configurational entropy, which stabilizes simple solid solution phases such as face-centered cubic (FCC), body-centered cubic (BCC), mixed structures over complex intermetallic compounds or hexagonal-close-packed (HCP) structure [2]. High-entropy alloys (HEAs) offer remarkable flexibility in alloy design and exhibit unique mechanical properties. In theory, all metallic elements and some non-metallic ones can be used to synthesize HEAs. However, certain elements are more commonly selected to form stable single-phase solid solutions, particularly those with simple face-centered cubic (FCC) or body-centered cubic (BCC) structures [3,4]. Elements such as Co, Cr, Fe, and Ni are typically used in FCC-structured HEAs, while Mo, Nb, Ta, and Zr are prevalent in refractory BCC-structured HEAs. HEAs with single solid-solution phases often display low hardness at room temperature, although some exhibit nano-twinning, which provides an excellent combination of strength and ductility at cryogenic temperatures.

On the other hand, HEAs containing intermetallic phases tend to offer higher hardness, but generally at the expense of ductility [5]. However, the vast number of possible elemental combinations and the complex interactions among these elements make it challenging to predict the performance of high-entropy alloys (HEAs). Even slight changes in composition can lead to significant differences in microstructure and mechanical properties. As a result, HEAs with different compositions may exhibit widely varying behavior, making experimental exploration both time-consuming and resource intensive. This variability underscores the importance of advanced predictive tools to efficiently navigate the compositional space and optimize alloy performance [6]. HEAs have demonstrated various superior properties, such as high strength and ductility, excellent wear and corrosion resistance, thermal stability, fracture toughness under extreme conditions, and hardness [7,8]. These properties arise from their high configurational entropy effect, sluggish diffusion, and lattice distortion [9]. These attributes make them attractive candidates for applications in aerospace, energy, defense, and nuclear sectors. However, the exceptional properties of HEAs are highly dependent on their composition, phase constitution, microstructure, and processing history. Among these, hardness is a critical property that reflects the material’s resistance to plastic deformation and often correlates with strength, elasticity, plasticity, toughness, and wear resistance [10]. In the extensive compositional space of high-entropy alloys (HEAs), the trial-and-error method is a widely used approach for identifying hardness. Although high-throughput experiments can significantly increase the screening time, they often lead to excessive raw material consumption and require sophisticated, high-cost equipment [11]. With the advancement of computational materials science, techniques such as density functional theory (DFT), molecular dynamics (MD), phase-field modeling, and CALPHAD-based phase diagram calculations, often combined with high-throughput strategies, have been increasingly applied to accelerate HEA design [12,13]. However, these computational methods are typically resource-intensive and time-consuming, especially when dealing with complex multicomponent systems. Therefore, enhancing computational efficiency and minimizing computational cost remain critical challenges in the field of HEA research.

In recent years, artificial neural networks (ANNs), a branch of machine learning (ML), have shown tremendous promise in modeling complex nonlinear relationships in materials science. ANNs are computational models inspired by the human brain, capable of learning from data and making accurate predictions even with limited physical modeling [14]. Various researchers have been focused on equiatomic CrMnFeCoNi high-entropy alloys [15,16,17]. These alloys show good resistance and excellent ductility. However, they mainly consist of a single-phase FCC structure, which results in low strength. This limits their use in structural applications that require both high strength and good wear resistance. Also, the high cost of Co makes these alloys expensive and less suitable for industrial use. To overcome these issues, researchers have added Al to FCC-type HEAs. Wang et al. [18] identified the mechanical properties of Al*_x_*CoCrFeNi alloy [19]. Chen et al. [20] studied the wear behavior of Al_0.6_CoCrFeNi at high temperatures. The alloy showed a dual-phase (FCC + BCC) structure with better wear resistance. Al also increased the hardness and improved the electrochemical properties. In addition, Al reduced the overall density, making the alloy more suitable for lightweight applications [9]. Inspired by the Cantor alloy (CoCrFeNiMn, single FCC phase) and Al*_x_*CoCrFeNi, this study focuses on the effect of AlCoCrFeMnNiW composition variation and sintering temperatures on hardness. Current trends emphasize explainable, physics-informed, and structure-aware models to improve extrapolation capability, accelerate design of superhard HEAs, and strengthen trust in computational predictions. In particular, machine learning (ML) has greatly advanced the atomic-scale design and characterization of HEAs by enabling accurate prediction of key nanostructural features such as grain size, phase formation, and defect evolution, thereby facilitating the optimization of their material properties [21]. Shrey Dixit et al. [22] used the Python program to calculate the physical and thermodynamic parameters such as entropy of mixing (ΔS_mix_), enthalpy of mixing (ΔH_mix_), atomic size difference (δ), the difference in electronegativity (χ), and average valance electronic configuration (VEC). These calculated parameters were further used to develop an artificial neural network (ANN) model with high prediction accuracy (87%). Nusrat Islam et al. [23] used five material parameters, such as the Pauling negativities (Δχ_P_), atomic size difference (δ), the entropy of mixing (ΔS_mix_), and mixing enthalpy (ΔH_mix_), to develop a backpropagation neural network (NN) to identify the amorphous structure, intermetallic compounds, and single-phase solid solution. The average generalization accuracy of the developed NN model is higher than 80%. Tancret et al. [24] developed a Gaussian process ML model based on physical parameters to design single SS HEAs. Huang et al. [25] used five empirical materials descriptors (ΔS_mix_, ΔH_mix_, δ, Δχ_p_, and VEC) and developed three different ML algorithms: K-nearest neighbors (KNN), support vector machine (SVM), and ANN with an accuracy of 68.6%, 64.3%, and 74.3%, respectively.

In this study, an ANN model was developed to accurately predict the hardness of AlCoCrFeMnNiW HEAs based on their elemental compositions and sintering temperature. Additionally, the ANN model was employed to identify optimal compositions and processing parameters for achieving enhanced hardness in HEAs. The influence of each input variable on hardness was evaluated using the Index of Relative Importance (IRI).

## 2. Materials and Methods

### Data Collection and Input-Output Variables of the Model

The artificial neural network (ANN) is a biologically inspired computational tool widely used to solve complex and nonlinear problems in various scientific and engineering fields [14]. An ANN mimics the function of the human brain by employing layers of interconnected processing units, known as neurons. These neurons are connected through weighted links that determine the influence of input signals on the output. The strength of these connections, known as weights, is adjusted during the training process to improve prediction accuracy. The ability of the ANN to generalize and learn from data makes it a powerful method for modeling problems that are difficult to solve using conventional analytical approaches. In this study, an ANN model was developed to predict the hardness of AlCoCrFeMnNiW HEAs based on their elemental composition, collected from the literature [26,27]. Among 535 data sets, 450 datasets were used for training the model, allowing it to learn the underlying patterns between composition and hardness. In comparison, the remaining 85 datasets were reserved for testing and validating the model’s predictive capability. The data set used in the current study is provided in the Supplementary Materials along with the ANN-predicted hardness. The last 85 data points in the Supplementary Materials (bold font) were used for testing the model, and the initial 450 data points were used for training. The compositional elements Al, Co, Cr, Cu, Mn, Ni, Fe, W, Mo, and Ti are considered input parameters, and hardness is considered as an output parameter. The training phase involves minimizing the error between predicted and actual values, typically through a backpropagation algorithm that adjusts the weights iteratively. The performance of the ANN is evaluated based on its ability to predict hardness with minimal deviation from the experimental data.

The artificial neural network (ANN) model was designed using a backpropagation algorithm combined with a sigmoid activation function [28]. The training process was executed using the C programming language, while a graphical user interface (GUI) was developed in Java to facilitate easy operation [29]. To achieve high predictive accuracy, the model’s hyperparameters, including the number of hidden layers, neurons per layer, learning rate, momentum, and iterations, were carefully optimized. The optimal network structure was selected based on the evaluation of root mean square error (RMSE) and average prediction error (Etr) values. Additionally, to ensure data uniformity and improve computational efficiency, all input and output variables were normalized within the range of 0.1 to 0.9 using a standard normalization equation, as presented below.

(1) $$y_{n}=\frac{\left(y-y_{min}\right)\times 0.8}{\left(y_{max}-y_{min}\right)}+0.1$$

where *y_n_* is the normalized value of *y*; *y_max_* and *y_min_* are the maximum and the minimum values of *y*, respectively. Once the optimally trained network was achieved, the normalized data were converted back to their original values using the following equation:

(2) $$y=\frac{\left(y_{n}-0.1\right)\left(y_{max}-y_{min}\right)}{0.8}+y_{min}$$

To achieve accurate predictions, the model underwent a rigorous training process aimed at minimizing the difference between predicted and experimental data. This process involved carefully adjusting key hyperparameters. The most suitable configuration was identified by selecting the setup that resulted in the minimum root mean square error (RMSE) and average prediction error (Etr).

(3) $$\mathrm{RMSE}=\frac{1}{P}\sum_{P}\sum_{i}{\left(T_{ip}-O_{ip}\right)}^{2}$$

(4) $$E_{tr}\left(x\right)=\frac{1}{N}\sum_{i=1}^{N}\left|\left(T_{i}\left(x\right)-O_{i}\left(x\right)\right)\right|$$

where *E_tr_*(x) is the average error in the prediction of training and testing data sets for output parameter x, *N* is the total number of data sets, *T_i_*(x) is the targeted output, and *O_i_*(x) is the output calculated [30].

## 3. Results and Discussion

### 3.1. Model Training

**Figure 1:**
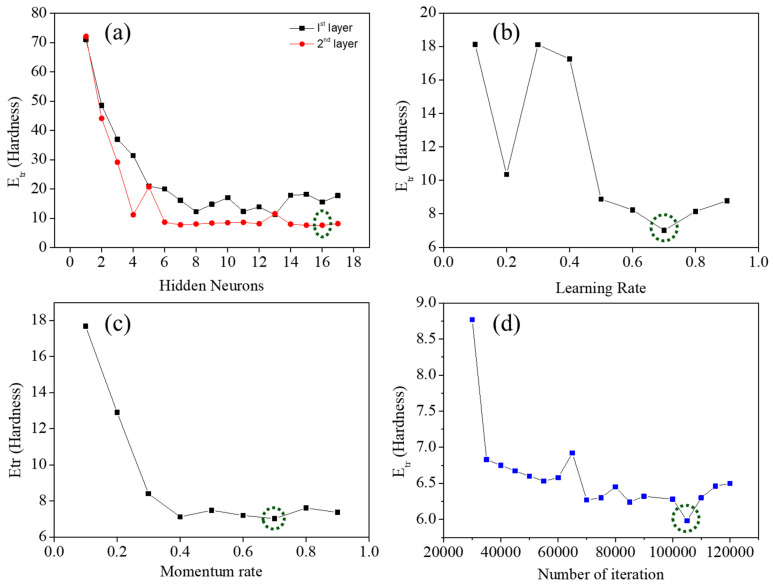
Variation in the average prediction error (Etr) with respect to different model parameters: (**a**) number of hidden neurons, (**b**) learning rate, (**c**) momentum rate, and (**d**) number of iterations.

The ANN model was trained to identify the most suitable network architecture and hyperparameter settings. The optimization process focused on minimizing the average prediction error (Etr), as illustrated in Figure 1. Both single-hidden-layer and two-hidden-layer network designs were systematically investigated. At the initial stage, a single-hidden-layer model was trained using a momentum rate of 0.5, a learning rate of 0.6, and 30,000 iterations, while the number of neurons in the hidden layer was gradually varied between 2 and 17. A similar approach was applied to a two-hidden-layer network. Among the tested configurations, the two-hidden-layer model with 16 neurons in each layer produced the lowest Etr. After identifying the optimal number of neurons, the iteration count was refined while keeping the momentum rate and learning rate fixed at 0.5 and 0.6 as shown in green circle, respectively. The number of iterations was adjusted within the range of 20,000 to 120,000, and the lowest error was recorded at 105,000 iterations, which was selected for subsequent training. In the final stage, the momentum and learning rates were systematically varied from 0.1 to 0.9 to achieve further optimization. The best performance was achieved with both parameters set to 0.7 [29,31].

### 3.2. Validation of the ANN Model

**Figure 2:**
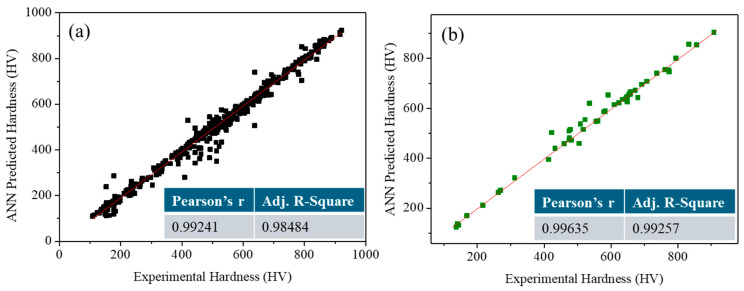
Validation of the ANN model: (**a**) training data and (**b**) test data of HEAs.

The performance of the developed model was evaluated using Pearson’s correlation coefficient (r) and the adjusted coefficient of determination (Adj. R^2^) between the experimental and predicted output values, as shown in Figure 2. The correlation coefficient values for both the training and testing datasets were found to be close to 1, indicating an excellent agreement between the predicted and experimental specific conductivity values. This high level of correlation confirms the strong predictive capability and reliability of the developed ANN model. We utilized the current model in various research areas, such as materials science and engineering [14,29,31,32]. A comparative analysis of the current model with other machine learning tools has been reported in our previous study [33].

The ANN model establishes the relationship between input and output variables through weighted connections, where each weight represents the strength of influence between neurons. During training, the model iteratively adjusts these weights to minimize prediction error and improve accuracy. The magnitude and direction of the weights determine the level of correlation between the input and output parameters [31].

### 3.3. Weight Distribution in the ANN Model

**Figure 3:**
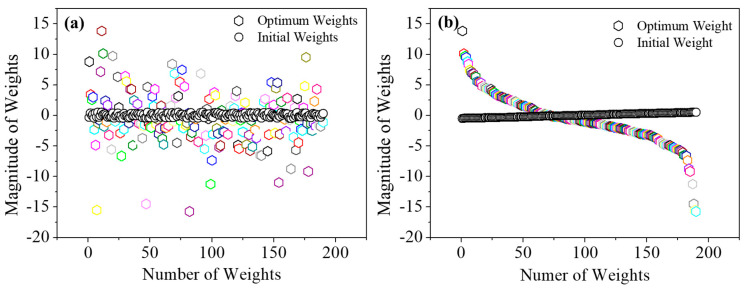
(**a**) Variation in the distribution of the weights at initial and optimum iteration. (**b**) Formation of sigmoid activation function at optimum weights.

In a neural network, artificial neurons are connected through adjustable parameters known as weights, which play a key role in information processing. These weights serve as the network’s memory, determining the strength and influence of each connection between the input and output layers [34,35]. As shown in Figure 3a, the distribution of weights evolves significantly during the training process. At the start of training (0 iterations), the weights are concentrated within a narrow range, approximately between −0.3 and +0.5. However, after 120,000 iterations, the range broadens substantially, spanning from −16 to +15. Figure 3b illustrates the behavior of a neuron using a sigmoid activation function. Initially, the weighted sum produces an almost linear response; however, as training progresses, the output curve develops into a characteristic sigmoidal shape. This progressive adjustment and structured distribution of weights enable the network to capture complex, nonlinear relationships within the data. As a result, the trained model can generate more accurate predictions and effectively generalize to unseen datasets.

### 3.4. Hardness Prediction

The AlCoCrFeNi high-entropy alloy (HEA) generally exhibits a dual-phase microstructure consisting of a face-centered cubic (FCC) phase along with either a body-centered cubic (BCC) or an ordered B2 phase. The phase composition is strongly influenced by the aluminum concentration and the processing route used [36]. When the Al content is relatively low, the FCC phase becomes predominant, resulting in improved ductility. Conversely, increasing the Al content favors the formation of harder, more brittle BCC or B2 phases, which are typically enriched with Al and Cr and tend to segregate within interdendritic regions or along grain boundaries. The FCC phase, generally rich in Fe, Ni, and Mn, contributes to ductility and forms the matrix in many cases, while the BCC phase, often enriched in Al, Cr, and W, provides high strength and hardness. The presence of tungsten (W), a high melting point and refractory element, promotes the formation of hard BCC or intermetallic phases such as Laves or σ phases, especially at grain boundaries or interdendritic regions [26]. These intermetallics can significantly enhance high-temperature strength and wear resistance but may reduce ductility. Due to the incorporation of large atomic radius W atoms, the lattice parameter increases (in FCC) or becomes irregular (in BCC). Increasing the tungsten (W) content in high entropy alloys (HEAs) significantly alters the microstructure by promoting severe lattice distortion. As W has a large atomic radius and low diffusivity, its addition increases the stability of BCC phases over FCC phases, leading to a transition from a ductile FCC or FCC + BCC structure to a harder and more brittle BCC-dominated or multiphase structure.

**Figure 4:**
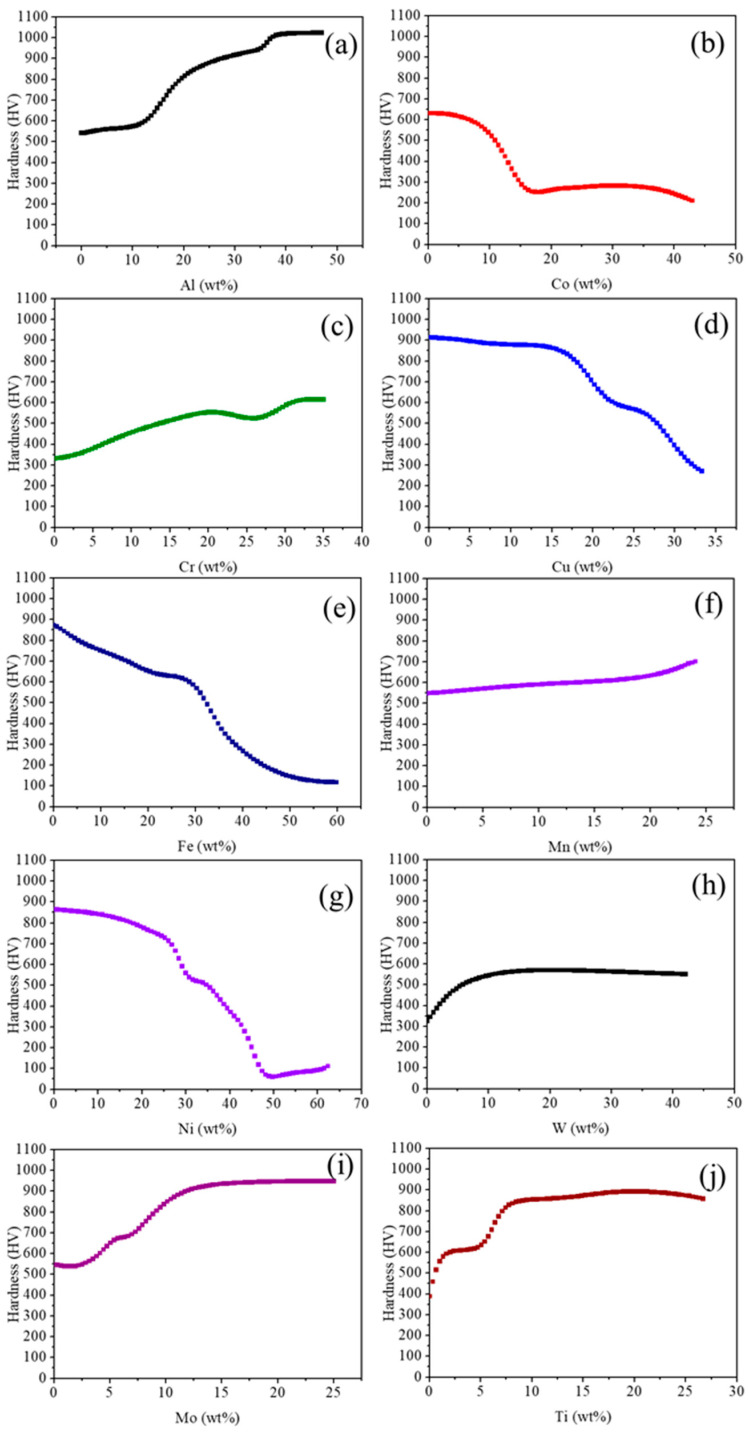
ANN predicted hardness as a function of individual element—(**a**) Al, (**b**) Co, (**c**) Cr, (**d**) Cu, (**e**) Fe, (**f**) Mn, (**g**) Ni, (**h**) W, (**i**) Mo, and (**j**) Ti.

The hardness of HEAs is directly influenced by their composition and microstructure. It was reported that the alloy composition determines the existing phases and their volume fractions, as well as atomic interactions that influence the properties through the intrinsic properties of the phases. However, the individual elemental effect on the hardness values of high-entropy alloys (HEAs) is rather limited, and only a few studies have focused on the variation in the effect of individual element amounts on the mechanical properties of HEAs [3]. So, in this study, we explained the impact of individual elements on the hardness with the help of a developed ANN model, as shown in Figure 4. Initially, we fixed the base composition at 5.4Al-17.83Cr-38.29Fe-21.97Mn-0W-0Co-0Cu-0Mo-0Ti. While varying the composition of an individual element, the contents of the other elements were kept constant. The selected element was varied systematically from its minimum to maximum range to study its effect on the Hardness.

The effect of Al elements shows (Figure 4a) the increment in the hardness with increasing composition. Similar trends were also found in a previous study [37]. The reason behind this is that as the Al content increases, the FCC phase transforms into a mixture of BCC and FCC phases, and eventually, at higher concentrations, a single-phase BCC and B2 structure develops. The BCC phase has a limited number of activated slip systems, which restricts plastic deformation and thus increases the hardness. The effect of Co (Figure 4b) on hardness shows a decrease in hardness with increasing composition. It is reported [38] that an increase in Co concentration promotes the formation of the FCC phase. The FCC phase possesses more ductility because FCC slip planes are more closely packed, reducing the atomic shear stress required for slip and lowering the activation energy for deformation. Another possible reason is that the Co has lower solubility in the BCC phase, which means Co is rejected from the BCC phase during solidification [33]. However, Cr shows an increase in hardness with rising concentration, as shown in Figure 4c. This is attributed to the fact that Cr stabilizes the BCC phase, leading to a higher BCC phase fraction, which in turn enhances the hardness [39]. As shown in Figure 4d, the effect of Cu shows a decreasing trend in the hardness with increasing Cu composition. It is found that with increasing Cu concentration, the BCC phases reduce and FCC phases increase, which encourages the flexibility of the alloy [40]. The effect of Fe on hardness is shown in Figure 4e. It is evident that increasing Fe content leads to a significant decrease in hardness. A similar trend was reported in a previous study [38]. Typically, the AlCoCrCuFeNi alloy exhibits a BCC structure along with the σ phase, identified as Cr_3_Ni_2_, which is inherently hard. However, as the Fe content increases, the fraction of the σ phase decreases, resulting in a reduction in overall hardness. However, another study [41] reveals that the hardness increases with Fe contents in Fe_x_Ni2Co2CrTiNb HEA. This improvement in hardness was attributed to the increased formation of the BCC phase at the expense of the FCC phase as the Fe content increased. As shown in Figure 4f, the hardness gradually increases with increasing Mn content. This enhancement in hardness can be attributed to solid solution strengthening and lattice distortion effects, arising from the significant atomic radius mismatch between Mn and the other alloying elements. The atomic radius of Mn (0.132 nm) is considerably smaller than that of Al (0.143 nm) and larger than those of the other major elements Co (0.126 nm), Cr (0.127 nm), Fe (0.127 nm), and Ni (0.124 nm) [42].

The hardness decreases with increasing Ni content up to 30 wt%, beyond which it remains relatively unchanged (Figure 4g). The decrease in the hardness is due to the dissolution of Cr and Fe precipitates in the nickel-rich matrix [43,44]. It is investigated that AlCrFeCoNix HEAs with low nickel content (≤1) predominantly exhibit a pure primitive cubic phase. As the Ni concentration increases, Ni begins to dissolve into the matrix, which facilitates the formation of an FCC phase [44]. This phase transformation leads to a reduction in hardness, as illustrated in Figure 4i, primarily due to the higher fraction of the softer FCC phase [45,46]. It also reported that the higher Ni concentrations lead to a significant reduction in hardness due to the stabilization of a single FCC phase [33]. Finally, the effect of W on hardness is shown in Figure 4h. The addition of W to the AlCoCrCuFeNi high-entropy alloy significantly increases its hardness. This enhancement is primarily attributed to the solid solution strengthening effect caused by the large atomic size mismatch between W and the other constituent elements. W atoms introduce severe lattice distortion, which impedes dislocation motion, thereby increasing the resistance to plastic deformation. Moreover, W has a high melting point and tends to form hard intermetallic compounds or secondary phases that contribute further to the hardness microstructure [26]. The effects of Mo and Ti are shown in Figure 4i and Figure 4j, respectively. Both increase the hardness and exhibit a similar trend on the hardness graph.

## 4. Conclusions

In this study, an artificial neural network (ANN) model was successfully employed to investigate the influence of individual elemental variations on the hardness of AlCoCrCuFeNi-based high-entropy alloys. The results revealed a strong correlation between alloy composition and hardness. Al and Cr were found to significantly enhance hardness by promoting the formation and stabilization of BCC and B2 phases, which are inherently harder due to limited slip systems. In contrast, elements such as Co, Cu, Fe, and Ni led to a reduction in hardness, primarily due to their role in stabilizing the ductile FCC phase or reducing the volume fraction of hard intermetallic phases. Mn contributed to hardness improvement through solid solution strengthening and lattice distortion effects. Notably, the addition of W markedly increased the hardness by inducing severe lattice distortion and promoting the formation of hard intermetallic compounds.

## Data Availability

The original contributions presented in this study are included in the article/Supplementary Material. Further inquiries can be directed to the corresponding authors.

## Supplementary Materials

The following supporting information can be downloaded at: https://www.mdpi.com/article/10.3390/ma18204655/s1.
