# Supplementary material for: Artificial Neural Network Approach for Hardness Prediction in High-Entropy Alloys
Source: Materials (Basel). 2025 Oct 10;18(20):4655. doi: 10.3390/ma18204655 (PMC12565321; doi:10.3390/ma18204655)
Supplement: Supplementary file 1 [file materials-18-04655-s001.zip › materials-3758690-supplementary.pdf]

Supplementary Material

Al	Cr	Fe	Mn	Ni	W	Co	Cu	Mo	Ti	Exp (HV)	ANN (HV)
5.4	17.83	38.29	21.97	16.77	0	0	0	0	0	420	419.968
7.78	19.99	21.47	0	50.77	0	0	0	0	0	315.7	310.744
10.61	20.45	6.59	0	62.34	0	0	0	0	0	415.7	410.988
10.68	20.58	18.79	0	49.95	0	0	0	0	0	559	554.313
10.28	19.81	21.27	20.93	0	3.5	0	24.21	0	0	680	677.4
9.93	19.14	20.56	20.43	0	6.56	0	23.39	0	0	705	701.926
7.84	15.11	16.22	15.96	0	26.41	0	18.46	0	0	780	778.108
0	23.06	24.77	0	26.03	0	26.14	0	0	0	570	541.025
7.49	28.88	31.02	0	32.6	0	0	0	0	0	552	547.966
10.49	20.21	21.7	0	0	0	22.9	24.7	0	0	770	766.952
0	17.99	19.32	0	20.31	0	20.39	21.99	0	0	400	394.074
7.95	15.32	16.45	16.19	17.29	26.8	0	0	0	0	461.26	454.166
0	18.5	23.96	20.33	19.41	0	17.8	0	0	0	352	346.069
0	18.5	23.96	20.33	19.41	0	17.8	0	0	0	424	412.742
10.9	21.01	59.79	0	8.3	0	0	0	0	0	580.3	571.18
10.86	20.93	22.48	22.11	23.62	0	0	0	0	0	552.7	546.628
10.47	20.18	20.67	21.32	22.78	3.59	0	0	0	0	502.92	495.578
10.11	19.49	20.93	20.59	22	6.89	0	0	0	0	490.15	482.335
13.17	21.15	22.71	0	42.97	0	0	0	0	0	543.9	534.683
21.41	22.92	24.62	0	31.05	0	0	0	0	0	638.6	631.126
0	0	23.56	0	24.76	0	24.86	26.81	0	0	330	324.253
0	0	24.45	24.05	25.7	0	25.8	0	0	0	500	491.154
0	0	24.45	24.05	25.7	0	25.8	0	0	0	460	447.976
0	0	24.45	24.05	25.7	0	25.8	0	0	0	350	342.041
6.17	11.89	12.77	12.57	0	42.06	0	14.54	0	0	891.5	888.286
10.86	20.93	22.48	22.11	23.62	0	0	0	0	0	753	745.961
10.47	20.18	21.68	21.32	22.78	3.57	0	0	0	0	644	640.142
10.13	19.53	20.97	20.63	22.04	6.69	0	0	0	0	923	919.228
27.22	24.28	26.08	0	21.93	0	0	0	0	0	738.4	736.066
35.77	26.01	27.94	0	10.28	0	0	0	0	0	842.7	832.2
0	23.06	24.77	0	26.03	0	26.14	0	0	0	490	541.025
13.6	26.21	28.15	0	0	0	0	32.03	0	0	650	646.178
9.3	25.6	27.5	0	28.9	0	8.7	0	0	0	624	622.932
10.65	20.53	22.05	21.69	0	0	0	25.09	0	0	633	631.14
10.47	20.7	30.01	0	38.55	0	0	0	0	0	559.6	556.357
10.8	20.8	40.22	0	28.18	0	0	0	0	0	510.2	510.179
10.84	20.9	49.38	0	18.87	0	0	0	0	0	537.2	538.478
6.69	21.52	23.11	0	24.29	0	24.39	0	0	0	594	597.665
0	23.06	24.77	0	26.03	0	26.14	0	0	0	590	591.301
0	0	23.56	0	24.76	0	24.86	26.81	0	0	385	383.311
0	0	23.56	0	24.76	0	24.86	26.81	0	0	340	339.703
7.59	15.32	16.46	16.19	17.29	26.79	0	0	0	0	839	840.631
10.5	20.23	21.72	0	22.83	0	0	24.72	0	0	704	781.72
10.5	20.23	21.72	0	22.83	0	0	24.72	0	0	851	781.72
8.78	16.92	18.17	17.87	19.09	0	19.17	0	0	0	662	663.251

0	0	32.18	0	33.82	0	33.99	0	0	0	380	388.643
10.5	20.23	21.72	0	22.83	0	0	24.72	0	0	879	878.496
2.59	16.84	18.09	0	19.01	0	22.87	20.59	0	0	645	645.799
10.5	20.23	21.72	0	22.83	0	0	24.72	0	0	443	441.427
0	23.06	24.77	0	26.03	0	26.14	0	0	0	400	406.422
0	23.06	24.77	0	26.03	0	26.14	0	0	0	570	541.025
0	18.54	19.92	19.59	20.93	0	21.02	0	0	0	646	644.533
0	22.2	22.2	0	22.2	0	22.2	0	11.1	0	220	220.528
10	30	20	0	20	0	20	0	10	0	440	439.458
20	20	20	0	20	0	10	0	10	0	801	797.845
20	20	10	0	20	0	20	0	10	0	755	750.861
19.6	19.6	11.8	0	19.6	0	19.6	0	9.8	0	754	772.075
20	20	20	0	10	0	20	0	10	0	708	701.812
16.7	16.7	16.7	0	25	0	16.7	0	8.3	0	586	589.787
15.4	15.4	15.4	0	30.8	0	15.4	0	7.7	0	395	393.362
16.7	16.7	16.7	0	16.7	0	25	0	8.3	0	741	740.879
0	23.5	23.5	0	23.5	0	0	5.9	0	23.5	825	829.942
0	22.2	22.2	0	22.2	0	0	11.1	0	22.2	727	723.101
0	21.1	21.1	0	21.1	0	0	15.8	0	21.1	661	659.794
0	20	20	0	20	0	0	20	0	20	657	649.609
0	20	20	0	20	0	20	0	0	20	887	868.137
15.2	15.2	15.2	0	30.3	0	0	24.2	0	0	372	362.542
4.8	23.8	0	0	23.8	0	0	23.8	0	23.8	376	370.34
20	20	0	0	20	0	0	20	0	20	513	505.769
0	0	25	0	20	0	20	0	25	0	458	456.861
20	0	20	0	20	0	20	0	20	0	750	744.594
2.5	22.5	25	0	25	0	25	0	0	0	135	140.447
5	20	25	0	25	0	25	0	0	0	147	140.096
7.5	17.5	25	0	25	0	25	0	0	0	175	170.375
10	15	25	0	25	0	20	0	0	0	190	197.806
12.5	12.5	25	0	25	0	25	0	0	0	210	209.378
15	10	25	0	25	0	25	0	0	0	250	259.286
17.5	7.5	25	0	25	0	25	0	0	0	314	315.51
20	5	25	0	25	0	20	0	0	0	386	376.895
22.5	2.5	25	0	25	0	25	0	0	0	486	427.52
20.6	15.9	15.9	0	31.7	0	15.9	0	0	0	340	375.61
18.2	18.2	18.2	0	18.2	0	0	18.2	0	9.1	460	450.095
15.4	15.4	15.4	0	15.4	0	0	15.4	0	23.1	596	590.341
20	20	20	0	20	0	0	0	0	20	687	677.458
22	18.3	18.3	0	18.3	0	18.3	0	0	4.6	666	646.963
21.1	17.5	17.5	0	17.5	0	17.5	0	0	8.8	717	703.916
20.2	16.8	16.8	0	16.8	0	16.8	0	0	12.6	702	697.209
19.4	16.1	16.1	0	16.1	0	16.1	0	0	16.1	676	666.299
16.7	16.7	16.7	0	16.7	0	16.7	0	16.7	0	851	847.488
9.5	19	19	0	19	0	19	14.3	0	0	240.7	246.743
10	20	20	0	20	0	20	10	0	0	240.8	231.595
13.5	19.2	19.2	0	19.2	0	19.2	9.6	0	0	312	332.023
14.3	19	19	0	19	0	19	9.5	0	0	341	336.687

15	20	20	0	20	0	20	5	0	0	375	371.647
15.2	19	19	0	19	0	19	8.6	0	0	403	406.599
17	18.9	18.9	0	18.9	0	18.9	18.9	0	0	560	538.795
19.6	19.6	19.6	0	19.6	0	19.6	19.6	0	0	578	559.107
19.2	19.2	19.2	0	19.2	0	19.2	0	0	3.8	467	464.246
16.7	16.7	16.7	0	16.7	0	0	33.3	0	0	375	363.419
23.8	23.8	4.8	0	23.8	0	23.8	0	0	0	637.2	679.969
3.5	17.5	17.5	0	26.3	0	26.3	0	0	8.8	480	474.854
3.2	16.1	16.1	0	24.2	0	24.2	0	0	16.1	671	661.778
18.2	18.2	18.2	0	0	0	18.2	9.1	0	18.2	804	790.911
17.4	17.4	17.4	0	0	0	17.4	13	0	17.4	613	605.516
16.7	16.7	16.7	0	0	0	16.7	16.7	0	16.7	582	575.785
5	25	0	0	25	0	22	22.5	0	0	165	176.163
0	24.4	24.4	0	24.4	0	24.4	0	2.4	0	150	154.73
0	20	40	0	20	0	20	0	0	0	124	123.247
0	14.3	57.1	0	14.3	0	14.3	0	0	0	109	111.612
0	16.7	50	0	16.7	0	16.7	0	0	0	115	113.649
13	21.7	21.7	0	21.7	0	21.7	0	0	0	245.8	283.554
4.8	23.8	23.8	0	23.8	0	23.8	0	0	0	169	138.684
25.9	18.5	18.5	0	18.5	0	18.5	0	0	0	516	511.15
13.3	0	0	0	33.3	0	33.3	20	0	0	193	200.494
7	23.3	23.3	0	23.3	0	23.3	0	0	0	166	161.956
8.6	22.9	22.9	0	22.9	0	22.9	0	0	0	131	193.804
16.7	16.7	16.7	0	16.7	0	16.7	16.7	0	0	472	455.702
16.7	16.7	33.3	0	16.7	0	16.7	0	0	0	485	475.822
16.7	0	16.7	0	16.7	0	16.7	16.7	0	16.7	626	622.353
16.7	16.7	16.7	0	33.3	0	0	16.7	0	0	396	399.869
16	20	20	0	20	0	20	0	0	4	473	473.007
18.2	18.2	18.2	0	18.2	0	18.2	0	9.1	0	747	747.081
18.2	18.2	18.2	0	18.2	0	18.2	0	0	9.1	507	480.375
10	20	20	0	20	0	20	0	0	10	520	520.232
23.89	19	19	0	19	0	19	0	0	0	493	503.481
25	0	25	0	25	0	25	0	0	0	447	433.543
26.5	14.7	14.7	0	29.4	0	0	14.7	0	0	570	568.03
27.3	18.2	18.2	0	18.2	0	18.2	0	0	0	510	515.764
28.6	14.3	14.3	0	14.3	0	14.3	0	0	14.3	729	666.612
20	20	20	0	0	0	20	20	0	0	407	385.877
20	0	20	0	20	0	20	20	0	0	536	529.451
0	16.7	16.7	0	25	0	25	0	0	16.7	596	635.008
0	25	25	0	25	0	25	0	0	0	156	150.987
0	18.2	18.2	0	27.3	0	27.3	0	0	9.1	447	442.651
34	34	5	0	10	0	17	0	0	0	699	688.958
47	15	13	0	6	0	14	5	0	0	728	760.927
38	6	31	0	5	0	11	9	0	0	604	593.074
36	31	9	0	8	0	10	6	0	0	790	790.487
43	15	14	0	11	0	17	0	0	0	806	759.354
47	12	13	0	11	0	12	5	0	0	811	778.761
43	19	11	0	8	0	19	0	0	0	855	838.215

39	31	8	0	7	0	10	5	0	0	802	776.62
47	13	12	0	10	0	13	5	0	0	799	796.419
43	22	8	0	7	0	20	0	0	0	857	848.218
47	19	10	0	5	0	14	5	0	0	855	823.062
43	19	9	0	9	0	20	0	0	0	816	806.217
46	23	7	0	5	0	14	5	0	0	811	794.469
44	22	9	0	6	0	14	5	0	0	790	797.338
43	24	8	0	6	0	19	0	0	0	855	837.812
43	24	10	0	5	0	18	0	0	0	856	820.277
47	20	9	0	5	0	14	5	0	0	859	827.89
43	22	5	0	6	0	24	0	0	0	875	835.71
43	21	8	0	5	0	23	0	0	0	863	857.598
47	20	11	0	5	0	12	5	0	0	843	815.67
43	17	14	0	6	0	15	5	0	0	794	768.976
42	30	7	0	6	0	10	5	0	0	783	760.636
47	22	7	0	5	0	11	5	0	0	797	776.561
47	20	10	0	5	0	13	5	0	0	805	821.599
29	34	5	0	10	0	22	0	0	0	699	685.242
47	16	16	0	5	0	7	9	0	0	749	722.101
43	21	12	0	5	0	14	5	0	0	828	802.877
47	15	10	0	5	0	18	5	0	0	849	805.335
47	18	5	0	5	0	20	5	0	0	883	854.089
43	20	12	0	7	0	18	0	0	0	864	843.748
47	16	10	0	5	0	17	5	0	0	841	804.619
43	23	5	0	5	0	24	0	0	0	868	852.675
43	22	5	0	5	0	25	0	0	0	865	845.489
46	18	5	0	5	0	21	5	0	0	840	853.862
43	22	8	0	5	0	22	0	0	0	882	858.471
43	23	7	0	5	0	22	0	0	0	883	856.773
43	21	9	0	7	0	20	0	0	0	828	848.55
43	25	5	0	5	0	22	0	0	0	848	846.339
47	19	5	0	5	0	19	5	0	0	878	856.888
43	18	5	0	5	0	29	0	0	0	782	774.337
9.1	22.7	0	0	21.7	0	0	22.7	0	22.7	392	375.923
13	21.7	0	0	21.7	0	0	21.7	0	21.7	452	436.62
20	20	20	0	20	0	20	0	0	0	350	483.952
16.7	16.7	16.7	0	16.7	0	16.7	0	0	16.7	435	434.737
23.8	23.8	23.8	0	5	0	23.8	0	0	0	615	626.875
25	25	25	0	0	0	25	0	0	0	720	757.713
23.8	23.8	23.8	0	23.8	0	5	0	0	0	550	570.656
22.5	22.5	22.5	0	22.5	0	10	0	0	0	539	559.557
16.7	16.7	16.7	0	16.7	0	33.3	0	0	0	532	516.62
23.8	5	23.8	0	23.8	0	23.8	0	0	0	438	451.005
22.5	10	22.5	0	22.5	0	22.5	0	0	0	476	493.874
21.3	15	21.3	0	21.3	0	21.3	0	0	0	510	507.776
16.7	33.3	16.7	0	16.7	0	16.7	0	0	0	617	632.532
25	25	0	0	25	0	25	0	0	0	712	740.287
23.8	23.8	5	0	23.8	0	23.8	0	0	0	665	673.752

22.5	22.5	10	0	22.5	0	22.5	0	0	0	587	614.174
21.3	21.3	15	0	21.3	0	21.3	0	0	0	558	589.355
15	28.3	28.3	0	0	0	28.3	0	0	0	655	653.736
20	26.7	26.7	0	0	0	26.7	0	0	0	695	706.614
40	20	20	0	0	0	20	0	0	0	775	780.375
5.7	18.9	18.9	0	18.9	0	18.9	18.9	0	0	183	185.966
13.8	17.2	17.2	0	17.2	0	17.2	17.2	0	0	272	301.836
20.6	15.9	15.9	0	15.9	0	15.9	15.9	0	0	473	498.545
26.5	14.7	14.7	0	14.7	0	14.7	14.7	0	0	604	595.374
23.1	15.4	15.4	0	15.4	0	15.4	15.4	0	0	508	545.009
31.5	13.7	13.7	0	13.7	0	13.7	13.7	0	0	602	630.522
33.3	13.3	13.3	0	13.3	0	13.3	13.3	0	0	623	653.983
35.9	12.8	12.8	0	12.8	0	12.8	12.8	0	0	653	692.181
37.5	12.5	12.5	0	12.5	0	12.5	12.5	0	0	688	687.384
2.4	24.4	24.4	0	24.4	0	24.4	0	0	0	118	159.896
14.9	21.3	21.3	0	21.3	0	21.3	0	0	0	338	408.292
16.7	20.8	20.8	0	20.8	0	20.8	0	0	0	382	425
18.4	20.4	20.4	0	20.4	0	20.4	0	0	0	527	560.375
31	17.2	17.2	0	17.2	0	17.2	0	0	0	482	494.563
14.3	0	14.3	0	14.3	0	42.9	14.3	0	0	166	183.741
23.8	0	23.8	0	23.8	0	4.8	23.8	0	0	531	545.385
22.2	0	22.2	0	22.2	0	11.1	22.2	0	0	545	559.896
18.2	0	18.2	0	18.2	0	27.3	18.2	0	0	366	376.776
16.7	23.5	16.7	0	16.7	0	33.3	16.7	0	0	249	262.128
5.9	21.1	23.5	0	23.5	0	23.5	0	0	0	110	144.073
15.8	20.5	21.1	0	21.1	0	21.1	0	0	0	388	452.596
17.9	19.2	20.5	0	20.5	0	20.5	0	0	0	538	581.536
3.8	19.2	19.2	0	38.5	0	0	19.2	0	0	162	160.098
5.7	18.9	18.9	0	37.7	0	0	18.9	0	0	170	182.192
7.4	18.5	18.5	0	37	0	0	18.5	0	0	200	215.987
9.1	18.2	18.2	0	36.4	0	0	18.2	0	0	238	257.999
10.7	17.9	17.9	0	35.7	0	0	17.9	0	0	278	296.378
12.3	17.5	17.5	0	35.1	0	0	17.5	0	0	290	320.437
13.8	17.2	17.2	0	34.5	0	0	17.2	0	0	315	337.113
15.3	16.9	16.9	0	33.9	0	0	16.9	0	0	339	358.531
19.4	16.1	16.1	0	32.3	0	0	16.1	0	0	521	557.461
23.1	15.4	15.4	0	30.8	0	0	15.4	0	0	546	587.092
24.2	15.2	15.2	0	30.3	0	0	15.2	0	0	550	570.887
28.6	14.3	14.3	0	28.6	0	0	14.3	0	0	567	572.607
30.6	13.9	13.9	0	27.8	0	0	13.9	0	0	576	583.627
33.3	13.3	13.3	0	26.7	0	0	13.3	0	0	593	607.767
21.7	20.8	21.7	0	13	0	0	21.7	0	0	495	519.438
20.8	19.2	20.8	0	16.7	0	20.8	20.8	0	0	486	502.935
19.2	18.5	19.2	0	23.1	0	19.2	19.2	0	0	408	430.944
18.5	16.7	18.5	0	25.9	0	18.5	18.5	0	0	369	366.749
16.7	20	16.7	0	25	0	16.7	8.3	0	0	358	394.344
20	25	10	0	20	0	20	10	0	0	586	623.219
16.7	15.4	16.7	0	16.7	0	16.7	8.3	0	0	601	619.178

15.4	0	15.4	0	15.4	0	30.8	7.7	0	0	295	325.829
22.2	15.4	22.2	0	22.2	0	22.2	11.1	0	0	584	569.063
15.4	16.7	15.4	0	30.8	0	15.4	7.7	0	0	310	356.113
16.7	10	16.7	0	16.7	0	25	8.3	0	0	451	488.729
20	30.8	20	0	20	0	20	10	0	0	546	557.27
15.4	16.7	15.4	0	15.4	0	15.4	7.7	0	0	607	623.366
16.7	15.4	25	0	16.7	0	16.7	8.3	0	0	537	546.992
15.4	20	30.8	0	15.4	0	15.4	7.7	0	0	514	529.667
20	22.2	20	0	10	0	20	10	0	0	604	632.7
22.2	22.2	22.2	0	0	0	22.2	11.1	0	0	639	658.114
22.2	15.4	22.2	0	22.2	0	22.2	11.1	0	0	534	569.063
30.8	20	15.4	0	15.4	0	15.4	7.7	0	0	609	633.657
20	22.2	20	0	20	0	10	10	0	0	551	560.303
11.1	22.2	22.2	0	22.2	0	0	22.2	0	0	382	398.268
27.3	18.2	18.2	0	18.2	0	0	18.2	0	0	573	589.323
33.3	16.7	16.7	0	16.7	0	0	16.7	0	0	651	661.035
7.7	0	30.8	0	30.8	0	30.8	0	0	0	137	137.085
14.3	0	28.6	0	28.6	0	28.6	0	0	0	209	220.149
20	0	26.7	0	26.7	0	26.7	0	0	0	383	394.73
8	17	17	0	33	0	17	8	0	0	280	311.88
23	23	15	0	16	0	15	8	0	0	580	598.078
11.8	29.4	44.1	0	14.7	0	0	0	0	0	450	455.49
6.3	15.4	46.9	0	15.6	0	31.3	0	0	0	304	323.465
38.5	14.3	15.4	0	15.4	0	15.4	0	0	0	695	579.704
42.9	14.3	14.3	0	14.3	0	14.3	0	0	0	740	637.644
28.6	20	14.3	0	14.3	0	14.3	14.3	0	0	571	560.253
20	16.7	0	0	20	0	20	20	0	0	415	405.69
25	20	16.7	0	16.7	0	16.7	8.3	0	0	602	600.51
0	20	20	0	20	0	20	20	0	0	144	174.009
43	33	6	0	6	0	6	6	0	0	764	775.502
42.9	7.1	7.1	0	21.4	0	14.3	7.1	0	0	591	560.138
42.9	7.1	7.1	0	14.3	0	21.4	7.1	0	0	701	654.232
40	6.7	20	0	6.7	0	13.3	13.3	0	0	768	759.374
46.2	7.7	15.4	0	7.7	0	15.4	7.7	0	0	702	670.318
42.9	14.3	7.1	0	14.3	0	14.3	7.1	0	0	720	687.627
42.9	14.3	14.3	0	14.3	0	7.1	7.1	0	0	694	660.371
7	23.3	23.3	0	46.5	0	23.3	0	0	0	149	164.655
11.1	22.2	22.2	0	44.4	0	0	0	0	0	229	239.289
13	28	6	0	31	0	0	22	0	0	459	474.438
12	31	5	0	31	0	0	21	0	0	469	471.553
12	31	5	0	32	0	0	20	0	0	483	465.296
10	35	5	0	25	0	0	25	0	0	472	454.258
10	35	5	0	24	0	0	26	0	0	454	463.655
10	35	5	0	26	0	0	24	0	0	441	443.33
11	29	5	0	26	0	0	29	0	0	495	485.402
11	28	5	0	25	0	0	29	0	0	477	483.077
11	28	6	0	28	0	0	27	0	0	469	466.786
17.2	17.2	17.2	0	17.2	0	17.2	0	0	13.8	525.2	489.263

0	24.4	24.4	0	24.4	0	0	2.4	0	24.4	834	820.968
0	0	35.51	0	32.68	0	31.81	0	0	0	125	130.659
9.63	0	30.45	0	29.19	0	30.73	0	0	0	138	163.028
17.42	0	27.81	0	27.28	0	27.49	0	0	0	212	209.26
22.87	0	26.13	0	25.15	0	25.85	0	0	0	385	414.547
28.42	0	24.38	0	23.28	0	23.92	0	0	0	453	447.583
0	22.2	22.2	0	22.2	0	22.2	0	11.2	0	218.5	220.357
10	20	20	0	20	0	20	0	10	0	414.4	444.819
18.2	18.2	18.2	0	18.2	0	18.2	0	9	0	717.5	747.933
25	16.7	16.7	0	16.7	0	16.7	0	8.2	0	650	663.433
30.8	15.4	15.4	0	15.4	0	15.4	0	7.6	0	602	609.498
0	18.7	19.3	0	27.1	0	27	0	0	9.3	509	515.749
3.8	17.6	17.1	0	26.3	0	26.1	0	0	9.1	487	477.481
0	17.1	16.3	0	24.6	0	24.5	0	0	17.5	654	659.517
3.9	15.6	15.6	0	23.6	0	23.9	0	0	17.4	717	731.86
17.5	25	25	0	25	0	7.5	0	0	0	624	643.685
0	20	20	0	20	0	20	20	0	0	286	174.009
0	20	20	0	20	0	0	20	20	0	263	265.026
20	20	20	0	20	0	0	20	0	0	342	458.85
20	20	20	0	20	0	20	0	0	0	395	483.952
27.2	18.2	18.2	0	18.2	0	18.2	0	0	0	402	515.697
33.2	16.7	16.7	0	16.7	0	16.7	0	0	0	432	475.771
38.4	15.4	15.4	0	15.4	0	15.4	0	0	0	487	582.422
42.8	14.3	14.3	0	14.3	0	14.3	0	0	0	506	636.111
20	20	20	0	20	0	20	0	0	0	520	483.952
19.6	19.6	11.8	0	19.6	0	19.6	0	9.8	0	750	772.075
18.2	18.2	18.2	0	18.2	0	18.2	0	0.91	0	730	776.815
16.7	16.7	25	0	16.7	0	16.7	0	8.3	0	634	653.436
15.4	15.4	30.8	0	15.4	0	15.4	0	7.7	0	634	650.366
0	20	20	0	20	0	20	20	0	0	137	174.009
5.66	18.87	18.87	0	18.87	0	18.87	18.87	0	0	178	186.098
9.09	18.18	18.18	0	18.18	0	18.18	18.18	0	0	207	223.666
13.79	17.24	17.24	0	17.24	0	17.24	17.24	0	0	270	298.24
16.67	16.67	16.67	0	16.67	0	16.67	16.67	0	0	406	455.694
20.63	15.87	15.87	0	15.87	0	15.87	15.87	0	0	475	498.985
23.08	15.38	15.38	0	15.38	0	15.38	15.38	0	0	510	544.297
26.47	14.71	14.71	0	14.71	0	14.71	14.71	0	0	560	595.078
28.57	14.29	14.29	0	14.29	0	14.29	14.29	0	0	568	616.384
31.51	13.7	13.7	0	13.7	0	13.7	13.7	0	0	604	630.609
33.33	13.33	13.33	0	13.33	0	13.33	13.33	0	0	625	653.871
35.9	12.82	12.82	0	12.82	0	12.82	12.82	0	0	660	691.999
37.5	12.5	12.5	0	12.5	0	12.5	12.5	0	0	643	687.384
25	25	25	0	25	0	0	0	0	0	472.4	489.441
23.81	23.81	23.81	0	23.81	0	0	0	4.76	0	548.5	560.084
22.22	22.22	22.22	0	22.22	0	0	0	11.11	0	621.5	626.829
20.83	20.83	20.83	0	20.83	0	0	0	16.67	0	853.8	858.512
20	20	20	0	20	0	0	0	20	0	914	912.955
0	22.22	22.22	0	22.22	0	22.22	11.11	0	0	166	176.766

9.09	18.18	18.18	0	18.18	0	18.18	18.18	0	0	225	223.666
8.77	17.54	17.54	0	17.54	0	17.54	17.54	0	3.51	272	269.585
8.47	16.95	16.95	0	16.95	0	16.95	16.95	0	6.78	321	313.185
8.2	16.39	16.39	0	16.39	0	16.39	16.39	0	9.84	458	459.43
7.94	15.87	15.87	0	15.87	0	15.87	15.87	0	12.7	588	591.614
7.69	15.38	15.38	0	15.38	0	15.38	15.38	0	15.38	635	638.798
7.46	14.93	14.93	0	14.93	0	14.93	14.93	0	17.91	650	650.601
7.25	14.49	14.49	0	14.49	0	14.49	14.49	0	20.29	654	656.893
7.04	14.08	14.08	0	14.08	0	14.08	14.08	0	22.54	661	665.244
6.85	13.7	13.7	0	13.7	0	13.7	13.7	0	24.66	669	680.307
6.67	13.33	13.33	0	13.33	0	13.33	13.33	0	26.67	697	694.181
9.09	18.18	18.18	0	18.18	0	18.18	18.18	0	0	223	223.666
20	0	20	0	20	0	20	20	0	0	532	529.451
16.67	16.67	16.67	0	16.67	0	16.67	16.67	0	0	468	455.694
16.67	0	16.67	0	16.67	0	16.67	16.67	0	16.67	624	620.101
16.67	16.67	16.67	0	16.67	0	0	16.67	0	16.67	468	471.2
0	25	25	0	25	0	25	0	0	0	156.6	150.987
23.81	23.81	23.81	0	23.81	0	0	0	0	4.76	643	641.521
0	25	25	0	25	0	25	0	0	0	131.8	150.987
11.11	22.22	22.22	0	22.22	0	22.22	0	0	0	206.2	238.198
0	25	35	0	25	0	0	0	15	0	567.3	555.287
0	26.05	25.09	0	24.4	0	24.47	0	0	0	113	159.612
5.88	24.49	23.82	0	22.59	0	23.21	0	0	0	113	145.63
8.03	23.44	23.22	0	22.69	0	22.63	0	0	0	196	188.353
12.1	21.55	21.66	0	22.06	0	22.63	0	0	0	209	244.774
15.66	21.54	20.92	0	20.69	0	21.19	0	0	0	280	432.51
17.33	20.93	20.18	0	20.43	0	21.15	0	0	0	361	417.51
20.12	20.7	20.33	0	19.27	0	19.58	0	0	0	433	464.228
25.53	19.06	19.11	0	19.06	0	19.24	0	0	0	499	519.677
27.44	18.26	17.95	0	17.66	0	18.69	0	0	0	517.3	527.027
30.13	17.96	17.29	0	16.97	0	17.65	0	0	0	512.4	523.958
28.57	14.29	14.29	0	14.29	0	14.29	0	0	14.29	643	664.75
20	20	20	0	0	0	10	0	10	0	801	811.182
22.22	22.22	22.22	0	0	0	22.22	0	11.11	0	796	843.486
20	20	20	0	0	0	30	0	10	0	741	759.822
17.86	17.86	17.86	0	0	0	35.71	0	10.71	0	586	617.138
0	20	20	0	20	0	20	20	0	0	119	174.009
10	18	18	0	18	0	18	18	0	0	203	237.113
16.7	16.7	16.7	0	16.7	0	16.7	16.7	0	0	410	455.702
23.08	15.384	15.384	0	15.384	0	15.384	15.384	0	0	504	544.36
28.5	14.3	14.3	0	14.3	0	14.3	14.3	0	0	575	615.768
33.5	13.3	13.3	0	13.3	0	13.3	13.3	0	0	612	657.302
37.5	12.5	12.5	0	12.5	0	12.5	12.5	0	0	640	687.384
21.74	21.74	21.74	0	13.04	0	0	21.74	0	0	496	525.479
20.83	20.83	20.83	0	16.68	0	0	20.83	0	0	486	493.811
20	20	20	0	20	0	0	20	0	0	495	458.85
19.23	19.23	19.23	0	23.08	0	0	19.23	0	0	407	424.976
18.52	18.52	18.52	0	25.92	0	0	18.52	0	0	367	396.633

25	0	0	0	25	0	0	25	0	25	537	515.806
22.5	0	15	0	20	0	0	20	0	22.5	516	524.021
20	0	20	0	20	0	0	20	0	20	516	489.96
25	0	0	0	25	0	0	25	0	25	521	515.806
22.5	0	15	0	20	0	0	20	0	22.5	434	524.021
20	0	20	0	20	0	0	20	0	20	365	489.96
25	0	0	0	25	0	0	25	0	25	518	515.806
22.5	0	15	0	20	0	0	20	0	22.5	575	524.021
20	0	20	0	20	0	0	20	0	20	544	489.96
25	0	0	0	25	0	0	25	0	25	416	515.806
22.5	0	15	0	20	0	0	20	0	22.5	516	524.021
20	0	20	0	20	0	0	20	0	20	449	489.96
20	20	20	0	20	0	20	0	0	0	543	483.952
9.4	22.7	22.7	0	22.7	0	22.7	0.0	0.0	0.0	178	208.561
12.5	21.9	21.9	0	21.9	0	21.9	0.0	0.0	0.0	272	263.622
15.6	21.1	21.1	0	21.1	0	21.1	0.0	0.0	0.0	529	452.641
18.8	20.3	20.3	0	20.3	0	20.3	0.0	0.0	0.0	531	552.069
21.9	19.5	19.5	0	19.5	0	19.5	0.0	0.0	0.0	527	482.421
25.0	18.8	18.8	0	18.8	0	18.8	0.0	0.0	0.0	517	508.56
9.1	18.2	18.2	0	18.2	0	18.2	18.2	0.0	0.0	225	223.16
8.7	17.5	17.5	0	17.5	0	17.5	17.5	0	3.5	275	270.047
8.4	16.9	16.9	0	16.9	0	16.9	16.9	0	6.7	320	312.619
8.1	16.3	16.3	0	16.3	0	16.3	16.3	0	9.8	455	462.879
7.9	15.8	15.8	0	15.8	0	15.8	15.8	0	12.6	590	607.067
7.6	15.3	15.3	0	15.3	0	15.3	15.3	0	15.3	635	645.845
7.4	14.9	14.9	0	14.9	0	14.9	14.9	0	17.9	650	651.737
7.2	14.4	14.4	0	14.4	0	14.4	14.4	0	20.2	655	657.52
7	14.1	14.1	0	14.1	0	14.1	14.1	0	22.5	660	665.054
6.8	13.7	13.7	0	13.7	0	13.7	13.7	0	24.6	665	679.995
6.6	13.3	13.3	0	13.3	0	13.3	13.3	0	26.6	700	694.088
0	25	25	0	25	0	25	0	0	0	238	150.987
11.11	22.22	22.22	0	22.22	0	22.22	0	0	0	262	238.198
0	25	25	0	25	0	25	0	0	0	160	150.987
13	23.5	20	0	23.5	0	20	0	0	0	386	377.473
0	25	25	0	25	0	25	0	0	0	134	150.987
0	18.18	18.18	0	27.27	0	27.27	0	0	9.09	442	440.399
0	22.2	22.2	0	22.2	0	22.2	0	11.1	0	220	220.528
10	30	20	0	20	0	20	0	10	0	440	439.458
20	20	20	0	20	0	10	0	10	0	801	797.845
15.4	15.4	15.4	0	15.4	0	30.8	0	7.7	0	586	594.774
16.7	16.7	25	0	16.7	0	16.7	0	8.3	0	635	653.436
15.4	15.4	30.8	0	15.4	0	15.4	0	7.7	0	639	650.366
25	16.7	16.7	0	16.7	0	16.7	0	8.3	0	665	667.124
30.8	15.4	15.4	0	15.4	0	15.4	0	7.7	0	615	610.399
22.2	22.2	22.2	0	0	0	22.2	0	11.1	0	857	842.713
23.8	23.8	23.8	0	23.8	0	0	0	4.8	0	549	560.63
22.2	22.2	22.2	0	22.2	0	0	0	11.1	0	622	626.522
20.8	20.8	20.8	0	20.8	0	0	0	16.7	0	854	860.694

20	20	20	0	20	0	0	0	20	0	905	912.955
0	25	25	0	0	0	25	25	0	0	134	142.351
17	23.3	23.3	0	0	0	23.3	23.3	0	0	180	172.18
11.1	22.2	22.2	0	0	0	22.2	22.2	0	0	207	201.386
16.7	20.8	20.8	0	0	0	20.8	20.8	0	0	271	275.972
24.5	18.9	18.9	0	0	0	18.9	18.9	0	0	476	484.988
27.3	18.2	18.2	0	0	0	18.2	18.8	0	0	510	516.052
31	17.2	17.2	0	0	0	17.2	17.2	0	0	557	569.701
33.3	16.7	16.7	0	0	0	16.7	16.7	0	0	567	588.935
36.5	15.9	15.9	0	0	0	15.9	15.9	0	0	603	614.361
38.5	15.4	15.4	0	0	0	15.4	15.4	0	0	624	632.115
41.2	14.7	14.7	0	0	0	14.7	14.7	0	0	657	658.153
42.9	14.3	14.3	0	0	0	14.3	14.3	0	0	644	652.954
0	22.2	22.2	0	22.2	0	22.2	11.1	0	0	172	176.222
8.8	17.5	17.25	0	17.5	0	17.5	17.5	0	3.5	272	274.095
8.5	16.9	16.9	0	16.9	0	16.9	16.9	0	16.9	321	359.437
8.2	16.4	16.4	0	16.4	0	16.4	16.4	0	9.8	458	457.388
7.9	15.9	15.9	0	15.9	0	15.9	15.9	0	12.7	590	592.661
7.7	15.4	15.4	0	15.4	0	15.4	15.4	0	15.4	636	637.371
7.5	14.9	14.9	0	14.9	0	14.9	14.9	0	17.9	646	650.446
7.2	14.5	14.5	0	14.5	0	14.5	14.5	0	20.3	664	657.097
7	14.1	14.1	0	14.1	0	14.1	14.1	0	22.5	657	665.054
6.8	13.7	13.7	0	13.7	0	13.7	13.7	0	14.7	667	665.921
6.7	13.3	13.3	0	13.3	0	13.3	13.3	0	26.7	696	694.4
0	20	20	0	20	0	0	20	20	0	263	265.026
18.2	18.2	18.2	0	18.2	0	9.1	18.2	0	0	473	487.295
18.2	9.1	18.2	0	18.2	0	18.2	18.2	0	0	367	377.907
18.2	18.2	9.1	0	18.2	0	18.2	18.2	0	0	418	418.352
18.2	18.2	18.2	0	9.1	0	18.2	18.2	0	0	423	435.324
5.3	21.1	26.3	0	26.3	0	21.1	0	0	0	168	169.065
12.5	12.5	12.5	0	50	0	12.5	0	0	0	225	225.818
**0**	**25**	**25**	**0**	**25**	**0**	**0**	**25**	**0**	**0**	**143**	**146.884**
**0**	**23.8**	**23.8**	**0**	**23.8**	**0**	**5**	**23.8**	**0**	**0**	**146**	**169.513**
**0**	**22.5**	**22.5**	**0**	**22.5**	**0**	**10**	**22.5**	**0**	**0**	**150**	**170.72**
**0**	**21.3**	**21.3**	**0**	**21.3**	**0**	**15**	**21.3**	**0**	**0**	**158**	**170.868**
**0**	**16.7**	**16.74**	**0**	**16.7**	**0**	**33.3**	**16.7**	**0**	**0**	**175**	**185.19**
**0**	**0**	**25**	**0**	**25**	**0**	**25**	**25**	**0**	**0**	**154**	**163.312**
**0**	**5**	**23.8**	**0**	**23.8**	**0**	**23.8**	**23.8**	**0**	**0**	**153**	**144.128**
**0**	**10**	**22.5**	**0**	**22.5**	**0**	**22.5**	**22.5**	**0**	**0**	**158**	**161.991**
**0**	**15**	**21.3**	**0**	**21.3**	**0**	**21.3**	**21.3**	**0**	**0**	**161**	**170.567**
**0**	**33.3**	**16.7**	**0**	**16.7**	**0**	**16.7**	**16.7**	**0**	**0**	**172**	**170.922**
**0**	**25**	**0**	**0**	**25**	**0**	**25**	**25**	**0**	**0**	**183**	**173.875**
**0**	**23.8**	**5**	**0**	**23.8**	**0**	**23.8**	**23.8**	**0**	**0**	**182**	**174.932**
**0**	**22.5**	**10**	**0**	**22.5**	**0**	**22.5**	**22.5**	**0**	**0**	**172**	**175.171**
**0**	**21.3**	**15**	**0**	**21.3**	**0**	**21.3**	**21.3**	**0**	**0**	**171**	**175.097**
**0**	**16.7**	**33.3**	**0**	**16.7**	**0**	**16.7**	**16.7**	**0**	**0**	**157**	**162.238**
**0**	**22.5**	**22.5**	**0**	**10**	**0**	**22.5**	**22.5**	**0**	**0**	**170**	**167.708**
**0**	**21.3**	**21.3**	**0**	**15**	**0**	**21.3**	**21.3**	**0**	**0**	**167**	**175.342**

0	16.7	16.7	0	33.3	0	16.7	16.7	0	0	158	161.433
0	26.7	26.7	0	20	0	26.7	0	0	0	140	156.797
0	20	20	0	40	0	20	0	0	0	125	128.735
14.3	14.3	14.3	0	42.9	0	14.3	0	0	0	242	242.149
15.4	15.4	15.4	0	38.5	0	15.4	0	0	0	265	273.157
16.7	16.7	16.7	0	33.3	0	16.7	0	0	0	335	350.531
0	28.3	28.3	0	15	0	28.3	0	0	0	170	176.784
5	31.7	31.7	0	0	0	31.7	0	0	0	475	493.564
10	30	30	0	0	0	30	0	0	0	620	635.671
18.2	18.2	18.2	0	27.3	0	18.2	0	0	0	503	498.971
21.3	21.3	21.3	0	15	0	21.3	0	0	0	555	542.273
22.5	22.5	22.5	0	10	0	22.5	0	0	0	548	574.922
0	0	33.3	0	33.4	0	33.3	0	0	0	125	135.94
7.7	0	30.1	0	30.1	0	30.1	0	0	0	138	137.355
14.2	0	28.6	0	28.6	0	28.6	0	0	0	212	224.066
20	0	0.27	0	0.26	0	0.27	0	0	0	385	391.759
0	25	25	0	25	0	25	0	0	0	131.8	150.987
0	18	18	0	27	0	27	0	0	10	509	512.521
0	16.7	16.7	0	25	0	25	0	0	16.6	654	633.695
10	20	20	0	20	0	20	0	10	0	440	444.819
20	20	20	0	20	0	10	0	10	0	801	797.845
20	20	10	0	20	0	20	0	10	0	755	750.861
19.6	19.6	12	0	19.6	0	19.6	0	9.6	0	754	772.436
20	20	20	0	10	0	20	0	10	0	708	701.812
18	18	18	0	18	0	18	0	10	0	747	742.797
16.7	16.7	16.7	0	25	0	16.7	0	8.2	0	586	586.979
15.4	15.3	15.3	0	30.1	0	15.4	0	7.7	0	395	398.229
16.7	16.7	16.7	0	16.6	0	25	0	8.3	0	741	740.13
15.4	15.4	15.4	0	15.4	0	30.8	0	7.6	0	586	587.624
16.7	16.7	25	0	16.6	0	16.7	0	8.3	0	635	655.957
15.4	15.4	30.7	0	15.4	0	15.4	0	7.7	0	639	653.815
25	16.7	16.7	0	16.7	0	16.7	0	8.2	0	655	663.433
30.8	15.4	15.4	0	15.4	0	15.4	0	7.6	0	615	609.498
3.5	17.5	17.5	0	26.3	0	26.3	0	0	8.9	480.8	482.639
3.2	16.1	16.1	0	24.2	0	24.2	0	0	16.1	671.6	661.778
18.2	18.2	18.2	0	18.2	0	18.2	0	0	9.1	460	480.375
28.5	14.3	14.3	0	14.3	0	14.3	0	0	14.3	643	663.236
22.2	22.2	22.2	0	0	0	22.2	0	11.2	0	857	841.485
25	25	25	0	25	0	0	0	0	0	472	489.441
24	24	24	0	24	0	0	0	4	0	549	547.48
22.2	22.2	22.2	0	22.2	0	0	0	11.2	0	622	627.586
20.8	20.8	20.8	0	20.8	0	0	0	16.7	0	854	860.694
20	20	20	0	20	0	0	0	20	0	905	912.955
8.7	17.5	17.5	0	17.5	0	17.5	17.5	0	3.8	272	272.306
8.5	16.5	16.5	0	16.5	0	16.5	16.5	0	6.7	321	331.735
8.2	16.4	16.4	0	16.4	0	16.4	16.4	0	9.8	458	457.388
7.9	15.9	15.9	0	15.8	0	15.9	15.9	0	12.7	590	593.993
7.7	15.4	15.4	0	15.4	0	15.4	15.4	0	15.3	636	637.9

7.5	14.9	14.9	0	14.9	0	14.9	14.9	0	17.9	646	650.446
7.2	14.5	14.5	0	14.5	0	14.5	14.5	0	20.3	664	657.097
7	14.1	14.1	0	14.1	0	14.1	14.1	0	22.5	657	665.054
6.8	13.7	13.7	0	13.7	0	13.7	13.7	0	24.7	667	680.651
6.7	13.3	13.3	0	13.3	0	13.3	13.3	0	26.7	696	694.4
16.7	0	16.7	0	16.7	0	16.7	16.7	0	16.5	626	628.934
0	20	20	0	20	0	0	20	20	0	263	265.026
22.5	0	15	0	20	0	0	20	0	22.5	516	524.021
20	0	20	0	20	0	0	20	0	20	516	489.96
25	0	0	0	25	0	0	25	0	25	537	515.806
